# The Long and the Short of It: Nanopore‐Based eDNA Metabarcoding of Marine Vertebrates Works; Sensitivity and Species‐Level Assignment Depend on Amplicon Lengths

**DOI:** 10.1111/1755-0998.14079

**Published:** 2025-02-10

**Authors:** Karlijn Doorenspleet, Lara Jansen, Saskia Oosterbroek, Pauline Kamermans, Oscar Bos, Erik Wurz, Albertinka Murk, Reindert Nijland

**Affiliations:** ^1^ Marine Animal Ecology Group Wageningen University and Research Wageningen the Netherlands; ^2^ Wageningen Marine Research Yerseke the Netherlands

**Keywords:** eDNA, fish, long read, metabarcoding, nanopore, North Sea

## Abstract

To monitor the effect of nature restoration projects in North Sea ecosystems, accurate and intensive biodiversity assessments are vital. DNA‐based techniques and especially environmental (e)DNA metabarcoding is becoming a powerful monitoring tool. However, current approaches rely on genetic target regions under 500 bp, offering limited taxonomic resolution. We developed a method for long‐read eDNA metabarcoding, using Nanopore sequencing of a longer amplicon and present DECONA, a read processing pipeline to enable improved identification of marine vertebrate species. We designed a universal primer pair targeting a 2 kb region of fish mitochondrial DNA and compared it to the commonly used MiFish primer pair targeting a ~ 170 bp region. In silico testing showed that 2 kb fragments improved accurate identification of closely related species. Analysing eDNA from a North Sea aquarium showed that sequences from both primer pairs could be assigned to most species, and additional species level assignments could be made through the 2 kb primer pair. Interestingly, this difference was opposite in eDNA from the North Sea, where not the 2 kb but the MiFish primer pair detected more species. This study demonstrates the feasibility of using long‐read metabarcoding for eDNA vertebrate biodiversity assessments. However, our findings suggests that longer fragments are less abundant in environmental settings, but not in aquarium settings, suggesting that longer fragments may provide a more recent snapshot of the community. Thus, long‐read metabarcoding can expand the molecular toolbox for biodiversity assessments by improving species‐level identification and may be especially useful when the temporal origin of the eDNA signal is better understood.

## 1 Introduction

North Sea fish populations are sensitive to disturbances such as fisheries, nutrient run‐off and increasing sea water temperatures (Andersen et al. 2020; Capuzzo et al. 2018; Hofstede et al. 2010; Krehenwinkel et al. 2019; O'Brien et al. 2019). Combined management strategies such as reduced fishing (Couce et al. 2020), designation of marine protected areas (MPA), and placing artificial hard substrates such as offshore wind parks are suggested to facilitate rehabilitation of the North Sea ecosystem (Claudet 2018; Degraer et al. 2020; Didderen et al. 2019; Kamermans et al. 2018). To understand how North Sea fish population dynamics are affected by these strategies, development and validation of methods that map fish population diversity and density is crucial. Conventional marine fish biomonitoring practices largely rely on destructive methods that involve netting and trapping (Daan et al. 2005; Reiss et al. 2010). These methods are costly, time‐consuming and require expert taxonomic visual identification skills (Mateos‐Rivera et al. 2020; Teletchea 2009). In addition, conventional methods have limited sampling efficiencies and may be disruptive to the environment (Eggleton et al. 2018). Thus, it is crucial to develop precise and non‐invasive biomonitoring solutions that are also time and cost efficient (Goodwin et al. 2017).

Environmental DNA (eDNA) based fish species identification has gained substantial attention in the last decade, as it can detect the presence of fish species based on a small amount of DNA present in seawater. It has been shown to be highly sensitive for non‐indigenous species detection (Ficetola et al. 2008) and identification of spawning and migration patterns (Thalinger et al. 2019). Short amplicon eDNA metabarcoding has become an increasingly popular tool to perform fish community assessment for identification of ecological relevant fish species from an array of ecosystems (Deiner et al. 2017; Miya et al. 2015; Ruppert et al. 2019; Taberlet et al. 2012; Thomsen et al. 2012). Also in the North Sea, species identified through metabarcoding of eDNA samples showed to be comparable to species caught in fyke nets in the same area (Bleijswijk et al. 2020).

The standardisation of eDNA metabarcoding as marine monitoring strategy is still under development. Species‐specific differences occur for example, in degree of skin cell shedding, degradation rates vary depending on temperature and season, and unknown dilution factors depending on currents all make quantification of the results challenging (Beng and Corlett 2020; Lacoursière‐Roussel et al. 2016; Sassoubre et al. 2016; Seymour et al. 2018). The sample preparation, metabarcoding technique and workflow will determine the quality of the results and thus the species detection quality and possible biases (Beng and Corlett 2020; van der Loos and Nijland 2021). Important steps in the protocol include decisions about methods of sampling and DNA extraction (Bessey et al. 2020; Hunter et al. 2005), primer and PCR settings (Doi et al. 2019; Sard et al. 2019; Zhang et al. 2020), sequencing technology (Egeter et al. 2020; Singer et al. 2019; Truelove et al. 2019), post‐sequencing data handling (Santos et al. 2020) and reference databases used (Hestetun et al. 2020; McGee et al. 2019).

Especially choice of primer pair and targeted DNA region are crucial for successful fish detection with eDNA (Beng and Corlett 2020). Several universal fish primer pairs are described and most target regions of the mitochondrial genome as there is a high copy number of this genome per cell (Schon 2000). The most used primer pairs target different short regions from 100 to 500 nucleotides of the 12S rRNA (Miya et al. 2015; Riaz et al. 2011; Taberlet et al. 2012), 16S rRNA (DiBattista et al. 2017; Evans et al. 2016), cytochrome B (Thomsen et al. 2012) and COI gene (Balasingham et al. 2018). Although primer pairs targeting short 12S regions are most used and considered as a standard (Shu et al. 2020), a longer target amplicon size facilitates distinguishing between closely related species and hence improves species level identification (Zhang et al. 2020). The use of multiple primer pairs is also suggested to increase taxonomic resolution (Evans et al. 2016; Miya et al. 2015; Zhang et al. 2020) and improved species level detectability has been demonstrated in lakes (Sard et al. 2019). Thus, using longer fragments and multiple markers can enhance the taxonomic resolution in metabarcoding studies.

Long read sequence analysis has been shown to be useful for species identification before in barcoding studies using Sanger sequencing (Hebert et al. 2003). Sanger sequencing accurately provides a DNA sequence from one individual but lacks the possibility of sequencing mixed communities like metabarcoding methods can (Kappel et al. 2017). On the other hand, commonly used Illumina platforms do not allow the use of long reads due to its ability to sequence with high accuracy but with a maximum read length of 500 bp (Tan et al. 2019). Fortunately, third generation sequencing as available from Oxford Nanopore Technologies (ONT) and Pacific Biosciences enables the generation of ultra‐long sequences and from mixed communities (Bleidorn 2016). This can be used for eDNA studies that are based on primer pairs targeting longer regions, covering several mitochondrial marker genes. This long amplicon approach was demonstrated to be successful in microbial metabarcoding studies and improved taxonomic assignment to species level (Johnson et al. 2019; Shin et al. 2016). Historically, the main limitation of nanopore sequencing was the large error rate of 5%–10% (Jain et al. 2015). This error rate can be overcome with bioinformatics tools to generate reliable consensus sequences and thus increase sequence accuracy (Baloğlu et al. 2021; Carradec et al. 2018; Egeter et al. 2022; Sahlin et al. 2021). To our knowledge, a bioinformatics pipeline that require little command‐line experience and generate a species list directly from raw sequence data from multiplexed metabarcoding experiments is not yet available for Nanopore short and long read metabarcoding. However, once installed, such a pipeline would greatly facilitate the development of DNA monitoring, as it also becomes feasible for non‐experts in bioinformatics.

This study assesses the utility of long and short read eDNA metabarcoding for fish and vertebrates using Oxford Nanopore sequencing. We present a new bioinformatics pipeline DECONA to analyse the obtained data, and we discuss the optimal settings of DECONA depending on amplicon length and sequencing chemistry. A new primer pair was developed, specific for fish and other vertebrates targeting a 2 kb fragment of both the 12S and 16S region of the mitochondrial rRNA genes. The primer pair was compared to the commonly used universal short read MiFish primer pair targeting a ~ 170 bp region of the 12S mitochondrial rRNA (Miya et al. 2015). We compared primer pairs *in silico* on several genetically similar species (< 3 nucleotide differences between species), to identify the discrimination power of each primer pair to species level. We also compared the sensitivity and taxonomic resolution resulting from both primer pairs using samples from a North Sea Ray Reef aquarium with a known species composition and field samples from distinct locations and habitat types in the North Sea. The DECONA pipeline was developed especially for Oxford Nanopore sequence data to increase the accuracy with which sequences can be assigned to species level and to reduce the bioinformatics skills required for analysis of the sequences.

## 2 Materials and Methods

### 2.1 Sample Collection—Ray Reef Aquarium

**FIGURE 1:**
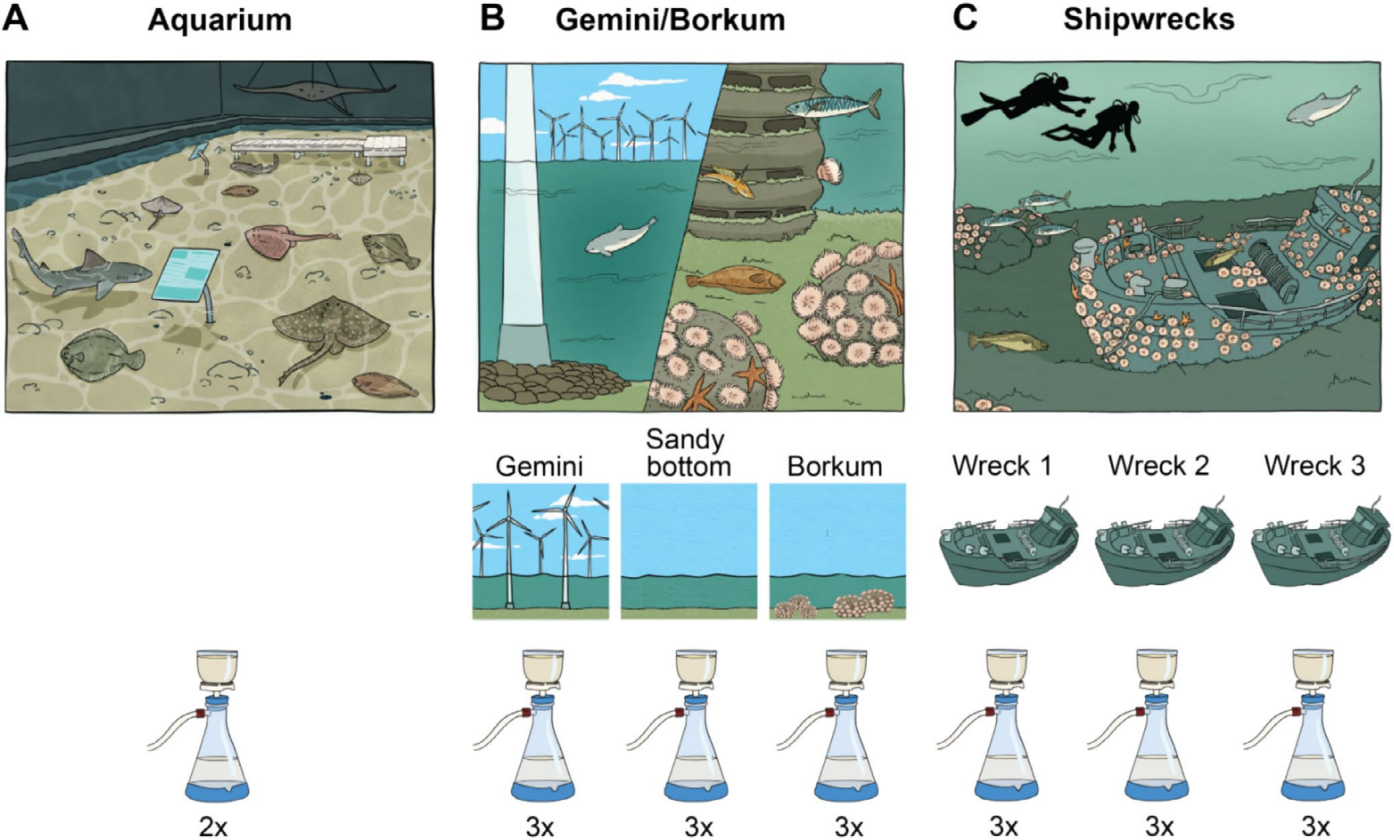
Sampling design of (A) North Sea “Ray Reef” aquarium, Dolfinarium, Harderwijk, the Netherlands. 2 × 1 L water just under the water surface using a 1 L plastic containers. (B) Borkum/Gemini where samples were taken in Gemini Wind Park, halfway between Gemini and Borkum on a sandy bottom and on the Borkum Reef Grounds. Seawater was collected using 2.5 L Niskin bottles. (C) North Sea shipwrecks with three different shipwreck locations where samples were taken near shipwrecks while diving, using an underwater pump with a balloon attached.

Two 1 L water samples were collected from the aquarium, just under the water surface using a 1 L plastic container pre‐sterilised with bleach (Figure 1A). One negative field control filter was taken by filtering demineralized tap water (Table S1). The aquarium has a volume of 200 m^3^ artificial seawater and represents a North Sea reef that contains bony fish, sharks, and rays with a total of 18 species (Table S2, for species list and abundance). The water temperature was 13°C, the salinity at 32.0‰ and the pH at 8.2 at the day of sampling.

### 2.2 Sample Collection—Gemini Wind Park/Borkum Reef Grounds

From Gemini Wind Park/Borkum Reef Grounds, samples were collected from inside Gemini Wind Park (54.0109 N, 6.0781E), halfway between Gemini Wind Park and the Borkum Reef Grounds on sandy substrate (53.8645 N, 6.2145E) (Sandy bottom) and at Borkum Reef Grounds (53.7016 N, 6.3467E using the WGS84 reference system). All samples were taken in July 2020 at slack tide during neap tides. Data on environmental parameters at the North Sea sampling locations were retrieved from the Copernicus Marine Service's Data Portal. Salinity varied in July 2020 between 31.8‰ and 34.4‰, temperature 15.7°C–18.6°C, and pH 7.9–8.1. Three 1 L replicates were collected at each location by sampling seawater using 2.5 L Niskin bottles at 0.5‐1 m above the seafloor (Figure 1B). One negative field control filter was taken by filtering demineralized tap water (Table S1).

### 2.3 Sample Collection—Shipwrecks

For the shipwrecks, samples were collected around three different shipwrecks in the North Sea while SCUBA diving in July 2019: wreck 1 (55.1821 N, 03.4446E) wreck 2 (55.2609 N, 03.5117E) and wreck 3 (55.0774 N, 02.5087E) (Figure 1C). On the sampling days and locations, salinity ranged between 34.2‰ and 34.27‰, temperature between 11°C and 12°C and pH was 8.06. Wreck 1 was sampled at 36 m depth, Wreck 2 at 32 m depth and wreck 3 at 30 m depth. At each sample location, three replicates of Several litres of water were collected at North Sea wreck sites near the bottom by scuba diving. The pump lever of a hand‐operated pump (ProPlus air & siphon pump 2‐in‐1 red, EAN 8717568798967) was operated 15‐20x to completely flush out the pump‐housing and tubing, before a punch balloon (Punch balloons, EAN 8021886316360) was attached to the outflow tube using a connector made of a 15 mL tube with the tip cut off. A 1 mm mesh was secured over the inflow tube with a rubber band. The inflow tube was held at the intended sampling site, and water was pumped in the balloon. The filled balloon was then clamped using two plastic sealing clips (BEVARA sealing clip, IKEA), and the balloon was stored in a mesh bag clipped to the diver's wing. One negative field control filter was taken by directly filtering tap water from a bottle and not from the decontaminated hand‐operated pump (Table S1).

### 2.4 Filtering Sample Water

All samples were immediately filtered using Thermo Scientific Nalgene Rapid‐Flow sterile disposable Filter Units CN (Cellulose nitrate) with a pore size of 0.8 μm. Filters were then individually placed in 2 mL screwcap Eppendorf tubes. The tubes were prefilled with 400 μL Zymo DNA/RNA shield (Zymo, USA) preservative. Samples were immediately stored at −20°C for a maximum of 1 month before further processing.

### 2.5 Primer Design

**FIGURE 2:**
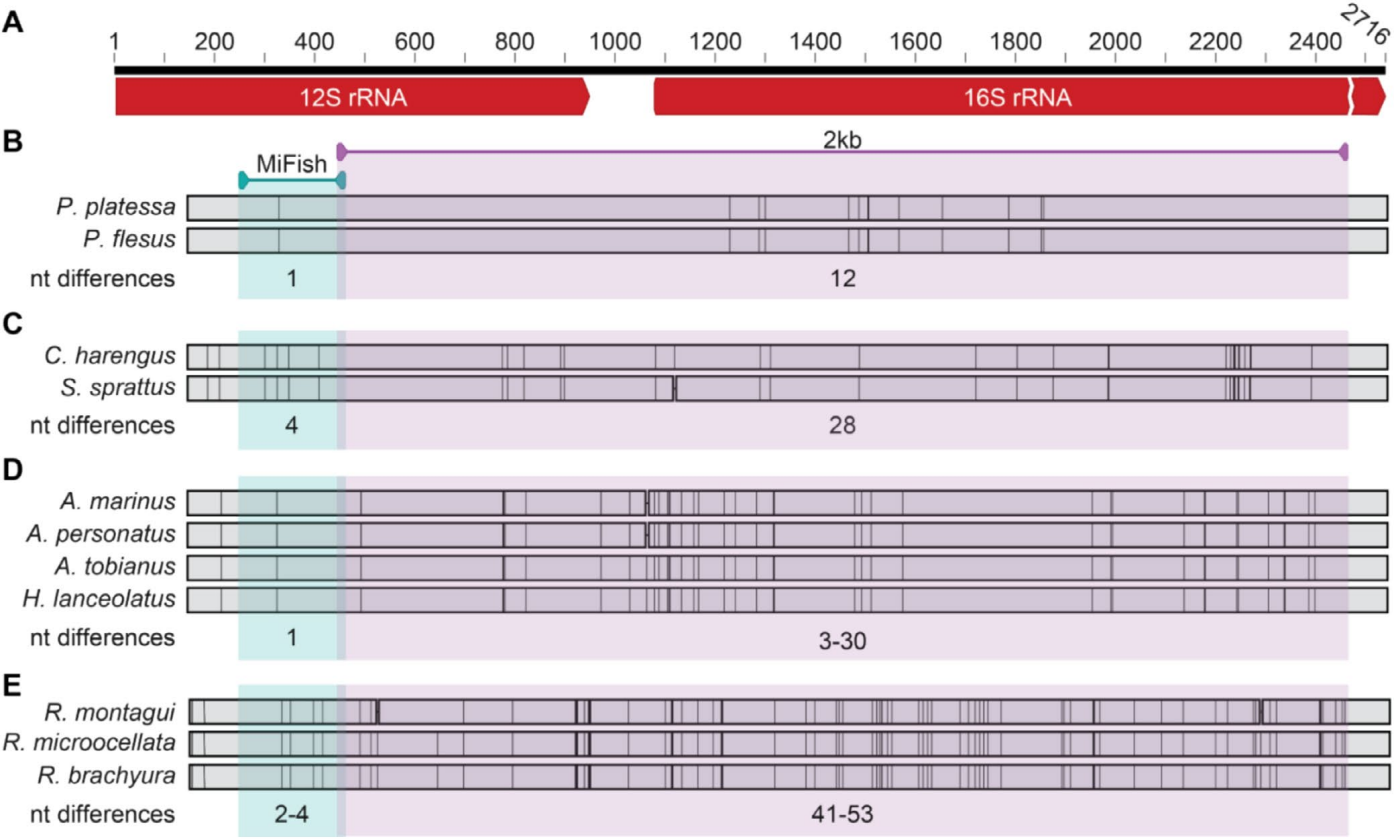
(A) The position of the new 2 kb primer pair (violet) and the MiFish primer pair (blue). (B) Genetic diversity between *Platichtys flesus* and *Pleuronectes platessa* for the target regions of the different primer pairs. The MiFish target region has 1 mismatch whereas the 2 kb region has 12 mismatches (C) *Sprattus sprattus* and *Clupea harengus*, where the MiFish target regions contains 4 mismatches and the 2 kb target regions contains 28 mismatches (D) four different *Ammodytes* species (*Ammodytes marinus, Ammodytes personatus, Ammodytes tobianus* and *Hyperoplus lanceolatus* ) with 1 mismatch in the Mifish target region and 3–30 mismatches in the 2 kb target region. (E) *Raja montagui, Raja microocellata and Raja brachyura*. Between 2–4 (MiFish) and 41–53 mismatches (2 kb). The (range of the) number of pairwise differences is indicated below each alignment.

Primer design is based on the adjacent ribosomal genes 12S and 16S of the mitochondrial genome of bony fish present in the North Sea according to a curated database of Dutch species (Naturalis Biodiversity Center, Nederlands soortenregister) consulted in 2019. The primer pair was designed *in silico* in Geneious prime 2019.0.4 (Kearse et al. 2012) and based on the NCBI available mitochondrial genomes of the target species (Table S3). A consensus sequence for each species was constructed when multiple genomes were available from the same species using default settings of the MAFFT alignment tool (v7.450, Katoh and Standley 2013) incorporated in Geneious. Consensus sequences of all species were aligned and forward and reverse primers was designed manually by locating regions with low genetic variation between target species. This resulted in a long read universal fish primer pair (Table 1) targeting a 2 kb fragment from ~450 bp downstream the start of the 12S rRNA gene in forward direction and ~ 300 bp upstream the end of the 16S rRNA gene (Figure 2A). The 5′ ends of the primers were extended with an ONT tag to allow for direct PCR based sample barcoding in downstream library preparation. To validate the 2 kb primer pair *in silico*, the primer pair was aligned against a curated North Sea database (see below) using Geneious prime 2023.0.4 (Kearse et al. 2012) in the “test with saved primers” mode (Primer3.2.3.7) allowing for 2 mismatches in the binding region. The primer pair was further validated with cutadapt v1.15 (Martin 2011) and showed that all mitochondrial sequences present in the database aligned with the primer pair in the target region.

**TABLE 1:** Primer sequences and characteristics of the newly designed forward and reverse primer for the 2 kb target region, including the ONT‐specific primer extension enabling PCR barcoding (Italics).

	Sequence	*T*_*m*_ ^a^	G/C content
Fish_12S_fw1‐ONT	*TTTCTGTTGGTGCTGATATTGC*GGATTAGATACCCYACTATGY	56.3°C—60.4°C	38.1%–47.6%
Fish_16S_rv1‐ONT	*ACTTGCCTGTCGCTCTATCTTC*GATTGCGCTGTTATCCCTRG	61.2°C—64.1°C	50%–55%

^a^ Calculations by ThermoFisher Scientific Tm calculator for Phire DNA polymerase, for the sequence specific part only.

### 2.6 In Silico Comparisons of Species Groups With Little Interspecific Genetic Differences

*In silico* comparative alignments were made from different taxonomic groups relevant for this study (e.g., sharks, rays, wrasses, gurnards, flatfishes, gobies, sand eels, mullets etc., data not shown) of (partial) mitochondrial references from the NCBI database. Genetically closely related species were aligned using Muscle 5.1 (Edgar 2004) multiple alignment tool in Geneious prime (Table S4, for accession numbers). Target regions of the different primer pairs were identified using “saved primers mode” (Primer3.2.3.7) allowing for two mismatches in the binding region.

### 2.7 DNA Extraction

Two different DNA extraction kits were used for different datasets due to the availability of kits in our lab at the time of processing. DNA from the aquarium samples was extracted using the DNeasy Blood & Tissue kit (Qiagen, USA). 20 μL Proteinase K was added to the samples in DNA/RNA shield, together with 400 μL lysis buffer and followed by 400 μL 70% ethanol. Further DNA extraction was performed using this kit following the protocol for tissue samples. DNA concentrations were measured using a Qubit 2.0 Fluorometer (Invitrogen, USA). DNA from filters from the two North Sea datasets were extracted using the Quick‐DNA miniprep kit (Zymo, USA) according to the manufacturer's instructions. Details of both protocols are also given at protocols.io (dx.doi.org/10.17504/protocols.io.6yfhftn).

### 2.8 Mock Community

To further test the test the primer design, DNA extracts of 12 North Sea fish species from different taxonomic groups were pooled in equimolar concentrations. This mock community contained: *Arnoglossus laterna, Chelon labrosus, Chelon ramada, Gadus morhua, Gasterosteus aculeatus, Petromyzon marinus, Neogobius melanostomus, Phycis blennoides, Salmo trutta, Scophthalmus rhomus, Solea solea* and *Trisopterus luscus* (Table S14). Of *Phycis blennoides, Scophthalmus rhomus and Petromyzon marinus*, no reference sequence is available, therefore these species were also separately amplified and sequenced, and the consensus sequence was used to correctly identify the species in the mock community.

### 2.9 Amplification

For PCR amplification of the samples with the 2 kb primer pair (aquarium, North Sea and shipwreck samples), 10 μL 2x Phire Tissue Direct PCR Master Mix (ThermoFisher Scientific, USA) was used. To the master mix 0.4 μL of each primer (10 mM), 0.5 μL eDNA template and nuclease free water (NFW) was added to a total of 20 μL. eDNA template was replaced with 0.5 μL NFW in case of PCR controls. Samples that were amplified with the MiFish primer pair (aquarium and North Sea) consisted of 5 μL 2x Phire Tissue Direct PCR Master Mix in combination with 1 μL template and 0.2 μL of each primer, and NFW added to a total of 10 μL. To reduce the effect of stochastic heterogeneity in PCR amplification, each sample was amplified using 3 PCR replicates. For the amplification with the 2 kb primer pair of the aquarium samples PCR settings were 98°C 180 s, 98°C 8 s sec, 57°C for 10 s, 72°C 30s, and 72°C 3 min with 36 cycles. For amplification with the MiFish primer pair, PCR settings were 98°C 180 s, 98°C 10 s, 59.6°C for 8, 72°C 10s, and 72°C 3 min with 35 cycles. PCR replicates were pooled prior to purification with SPRI magnetic beads (2:1 ratio).

### 2.10 Nanopore Sequencing

All samples were barcoded using the PCR barcoding kit 96 (EXP‐PCB096), and sequencing libraries were created using the SQK‐LSK114 kit (Oxford Nanopore Technologies Ltd., UK). The following adaptations were made from the manufacturer's instructions: barcoding PCR was performed in a total volume of 15 μL containing 0.3 μL PCR barcode primer pair and 10‐50 ng amplicon. The applied barcode PCR program was as follows: initial denaturation at 95°C for 180 s, 15 cycles of 95°C for 15 s, 62°C for 15 s (10s for MiFish), 65°C for 90s, followed by a final extension at 65°C for 180 s. A negative control was taken along in which NFW was added instead of first‐round PCR amplicon template. After the barcoding PCR, sample concentration was estimated using the Qubit HS kit on the non‐purified barcoded PCR products, and samples were pooled in equimolar ratios. The pooled amplicon sequence library was cleaned using SPRI magnetic beads, washed once with freshly prepared 70% ethanol and once with a 2:1 mixture of Long Fragment Buffer (LFB) and Short Fragment Buffer (SFB) (LFB and SFB are supplied with the Ligation sequencing kits from ONT) to enrich for the 2 kb target size fragments, and only SFB for MiFish samples. During final clean‐up, the library was again washed in a 2:1 mixture of LFB and SFB (2 kb) or SFB only (MiFish). A maximum of 100 ng DNA was loaded on a primed flow cell to prevent overloading of the flow cell. For the 2 kb samples, sequencing was performed until a sequencing depth of 4,082,320 reads. MiFish samples were sequenced with a sequencing depth of 2.059.490 reads per barcode for aquarium and Gemini wind park/Borkum reef ground samples and 15.046.781 reads from Wreck samples. Sequencing was performed with a R10.4.1 flow cell on an Oxford Nanopore MinION Mk1C device with a sequencing speed of 450 bases per second. For all PCRs, negative controls were considered (Table S1).

### 2.11 Sequence Read Processing With DECONA

To process sequencing data and generate consensus sequences from mixed samples we designed a bioinformatics pipeline called DECONA (https://github.com/Saskia‐Oosterbroek/DECONA). This pipeline clusters Nanopore reads, aligns them, creates a consensus sequence, and has the option to apply ONT specific polishing. The DECONA pipeline takes fastq files as input. The DECONA pipeline starts with filtering the fastq files on desired length and quality score with Nanofilt v2.8.0 (De Coster et al. 2018). Then, cutadapt v4.8 (Martin 2011) is optionally used to trim primer sequences from the reads. CD‐hit v4.8.1, a program that clusters reads based on short words rather than sequence alignment is used to cluster the reads based on a set percentage of similarity (W. Li et al. 2002). The clustered reads are subsequently aligned using Minimap2 v2.21 (H. Li 2018). Based on these alignments, Racon v1.4.20 is used to build the initial draft consensus sequence of each cluster (Vaser et al. 2017) which is then optionally polished by Medaka v1.4.3 (Oxford Nanopore Technologies Ltd., UK).

In this research, base‐calling of the raw fast5 files was performed using Guppy (Version 6.5.7, Oxford Nanopore Technologies Ltd., UK) in super high accuracy (SUP) mode for the MiFish samples of Aquarium and Gemini/Borkum reef ground. For all 2 kb and MiFish samples of the Wreck dataset, basecalling of pod5 files was performed using Dorado (Version 0.8.1, Oxford Nanopore Technologies Ltd., UK) After this, DECONA1.5 was used to filter the fragments of the 2 kb primer pair dataset at 1800–2350 bases and cluster these reads at 95% similarity. For the fragments of the MiFish primer pair dataset filtering was set between 160 and 240 bases and clustering at 97% similarity. All data were filtered at the default quality score of Q10. Large clusters were set to be randomly subsampled to a maximum cluster size of 500 reads. Medaka polished consensus sequences were created from each cluster larger than 5 reads. Initial polished consensus sequences were re‐clustered at 99%. The commands used to run DECONA were as follows:

2 kb R10: decona ‐f ‐T 32 ‐l 1800 ‐m 2350 ‐g “GGATTAGATACCCYACTATGY;max_error_rate = 0.1;min_overlap = 17 … CYAGGGATAACAGCGCAATC;max_error_rate = 0.1;min_overlap = 17” ‐n 10 ‐r ‐o 0.99 ‐R 500 ‐k 6 ‐M ‐c 0.95 ‐b /home/reindert/Blast_database/eDNA_NZ_23/North_sea_232.

MiFish R10: decona ‐f ‐T 32 ‐l 160 ‐m 240 ‐g “GTYGGTAAAWCTCGTGCCAGC;max_error_rate = 0.1;min_overlap = 20 … CAAACTYGGATTAGATACCCCACTAT;max_error_rate = 0.1;min_overlap = 20” ‐n 10 ‐r ‐o 0.99 ‐R 500 ‐k 6 ‐M ‐c 0.97 ‐b /home/reindert/Blast_database/eDNA_NZ_23/North_sea_232.

DECONA settings.

Different combinations of raw read *Q*‐score (−q) and cluster similarity (−c) settings were tested to determine their optimal use in various laboratory choices. Samples from the ray reef aquarium, including both 2 kb and MiFish samples, were utilised to assess optimal settings for different amplicon target lengths. Additionally, various cluster similarity settings were tested on the wreck samples to determine optimal settings for different sequencing chemistries. Optimal settings were determined by running all combinations of cluster similarities (0.80, 0.85, 0.90, 0.95, 0.97, 0.99 (2 kb only), and 1) and Q‐scores (8, 10, 12, 15, 17, and 20) in looped DECONA runs. Total reads, consensus sequences (clusters), and identified species were recorded for each setting. Based on the highest number of species found, the optimal settings were chosen for the consensus building with DECONA.

### 2.12 Curated North Sea Fish Reference Database Building

For taxonomic identification, an in‐house reference database was compiled based on whole mitochondrial genome sequences available in the NCBI database for North Sea fish species (last search October 2024, Table S5). When the whole mitochondrial genome was not available, available sequences of (fragments of) the 12S and/or the 16S rRNA genes from these species were added to the database. To validate correct species identification, closely related species that do not occur in the North Sea were also added to the database. Although our primer design was based on mitogenomes of bony fish, the resulting primer pair turned out to be universal not only to bony fish, but also to elasmobranchs and other marine vertebrates. Therefore, these taxonomic groups were also added to the database. In addition, frequently occurring contaminants as of human, chicken, cow, and pig were added to the database to prevent a large portion of unidentified reads resulting from contamination. The complete database consisted of 536 sequences of which 113 were complete mitogenomes and 30 were complete 12S and 16S regions (Table S5). The database contained 222 unique species.

### 2.13 Taxonomic Assignment of Consensus Sequences

The BLASTn (NCBI, version 2.11.0) function that was built within the DECONA environment was used against our North Sea fish reference database for taxonomic assignment of the consensus sequences derived from DECONA. To automate further assessment of the BLASTn output for accurate species‐level identification, a script was developed in R studio (2022.12.0) and integrated into DECONA. This script can be found at github.com/karlijn‐doorenspleet/decona‐postprocessing/. This script retrieved the taxonomic lineage from NCBI using *taxize* (v0.9.96, Chamberlain and Szöcs 2013). The top five hits were considered based on the highest e‐value for each consensus sequence. Within each top five hit, sequence was labelled as *unclassified* on species level if they had the same e‐value, percentage identity and alignment length but did not share the same taxon (species, genus, family, order, class, phylum). After that, the top hit (based on e‐value) was kept for further quality threshold control. As such, species that shared the same sequence and had a high similarity hit with the reference database, were excluded from species level taxonomic assignment to avoid misidentifications (for examples of such cases, see Table S6). Of all the taxonomically assigned sequences, top hits were considered for species level assignment, based on specific thresholds per amplicon length. For the 2 kb fragment the thresholds were a minimal alignment length of 1100 nucleotides with < 30 mismatches and > 98% identity for species level assignments. Hits with percentage identities > 97% sequences were assigned on genus level and with > 95% sequences were assigned at family level but not further considered for this study. The following thresholds were considered based for the MiFish fragment: < 4 mismatches, > 98% identity, and a minimal alignment length of 100 nucleotides. Assignments that did not meet these thresholds were renamed to *unclassified* and this was applied to both 2 kb and MiFish fragments. The finding of *Cheliyoditchus lucerna* in the ray reef aquarium (2 kb) and *Molva molva* in the Gemini/Borkum dataset (2 kb) are indicated with a star, as for these findings a species level assignment of *Cheliyoditchus kumu* and *Molva dypterygia* was found respectively. The assumingly correct species was reported with a star, as incorrect species identification happened due to the lack of 12‐16S fragments of the North Sea species in the database.

### 2.14 Analysis of Taxonomic Assignments

Rarefaction curves were plotted (vegan package, v 2.6–4, Table S7) and showed flatting curves, indicating that enough sequencing depth was reached for all samples, and no samples were further rarefied or removed. Sequence abundance was log10 transformed for all datasets. Reads classified as belonging to the genera *Homo*, *Ovis*, *Gallus*, *Bos* and other non‐marine animals were set to *unclassified*, along with all consensus sequence that did not have a hit with a database on species level (see Table S8 for the read percentage of non‐target hits per barcode). For alpha diversity, both Shannon indices and observed values were calculated and were tested using Shapiro–Wilk for normal distribution of the data, two‐way ANOVA to test for significant differences between alpha diversities, primer pair and location, and post hoc Tuckey HSD test for pairwise comparison. For beta diversity, non‐metric multidimensional scaling (‘bray’) was performed in combination with *betadisper* to check for homogeneity of variance and PERMANOVA to analyse the effect of treatments between samples (adonis, vegan). Post hoc analysis was performed using the *pairwise.adonis* package in combination with *devtools* when applicable. Sequencing of control samples and PCR controls resulted no reads at all or, non‐target species (e.g., *Homo sapiens* ) in all control samples. Only the Wreck MiFish control samples, that contained *Pomatoschistus microps* which did not occur in any other samples. Control samples were therefore excluded from further analysis (Table S1).

### 2.15 In Silico Comparisons of Species Groups With Little Interspecific Genetic Differences

*In silico* comparative alignments were made from different taxonomic groups relevant for this study (e.g., sharks, rays, wrasses, gurnards, flatfishes, gobies, sand eels, mullets etc., data not shown) of (partial) mitochondrial references from the NCBI database. Genetically closely related species were aligned using Muscle 5.1 (Edgar 2004) multiple alignment tool in Geneious prime (Table S3, for accession numbers). Target regions of the different primer pairs were identified using “saved primers mode” (Primer3.2.3.7) allowing for two mismatches in the binding region.

## 3 Results

### 3.1 *In Silico* Comparison of Primer Pair Performance on Closely Related Taxa

*In silico* alignments show *Pleuronectes platessa* and *Platichthys flesus* target regions differ 1 nucleotide when using the MiFish primer pair (99.4% similarity) whereas the target region of the 2 kb primer pair has 12 nucleotide differences (99.3% similarity) (Figure 2B). *Clupea harengus* and *Sprattus sprattus* diverged by 4 nucleotides (98.3% similarity) in the MiFish target region and their 2 kb target region showed a pattern of 29 nucleotide differences (98.6% similarity) (Figure 2C). Sand eel species *Ammodytes marinus* , *Ammodytes personatus* , *Ammodytes tobianus* and *Hyperoplus lanceolatus* also show 1 nucleotide mismatch (99.6% similarity) between all species for the MiFish target region. From the 2 kb target region, *Ammodytes marinus* differed from *Ammodytes tobianus* and *Hyperoplus lanceolatus* with 29 and 30 nucleotide differences, respectively (98.3% similarity) whereas between *Ammodytes tobianus* and *Hyperoplus lanceolatus* the genetic diversity remains low with 3 nucleotide differences (99.8% similarity) (Figure 2D). *Raja brachyura* and *Raja microocellata* showed 4 nucleotide differences for the MiFish fragment (98.3% similarity), while *Raja montagui* differed 2 nucleotides with from both *Raja microocellata and Raja brachyura* (99.1% similarity). Nucleotide differences greatly increase when comparing the 2 kb region: 41 nucleotide differences between *Raja brachyura* and *Raja microocellata* (97.7% similarity), 47 between *Raja brachyura* and *Raja montagui* (98% similarity), and finally 53 between *Raja microocellata and Raja montagui* (97.4% similarity) (Figure 2E).

### 3.2 Optimal DECONA Settings Are Different per Primer Pair

**FIGURE 3:**
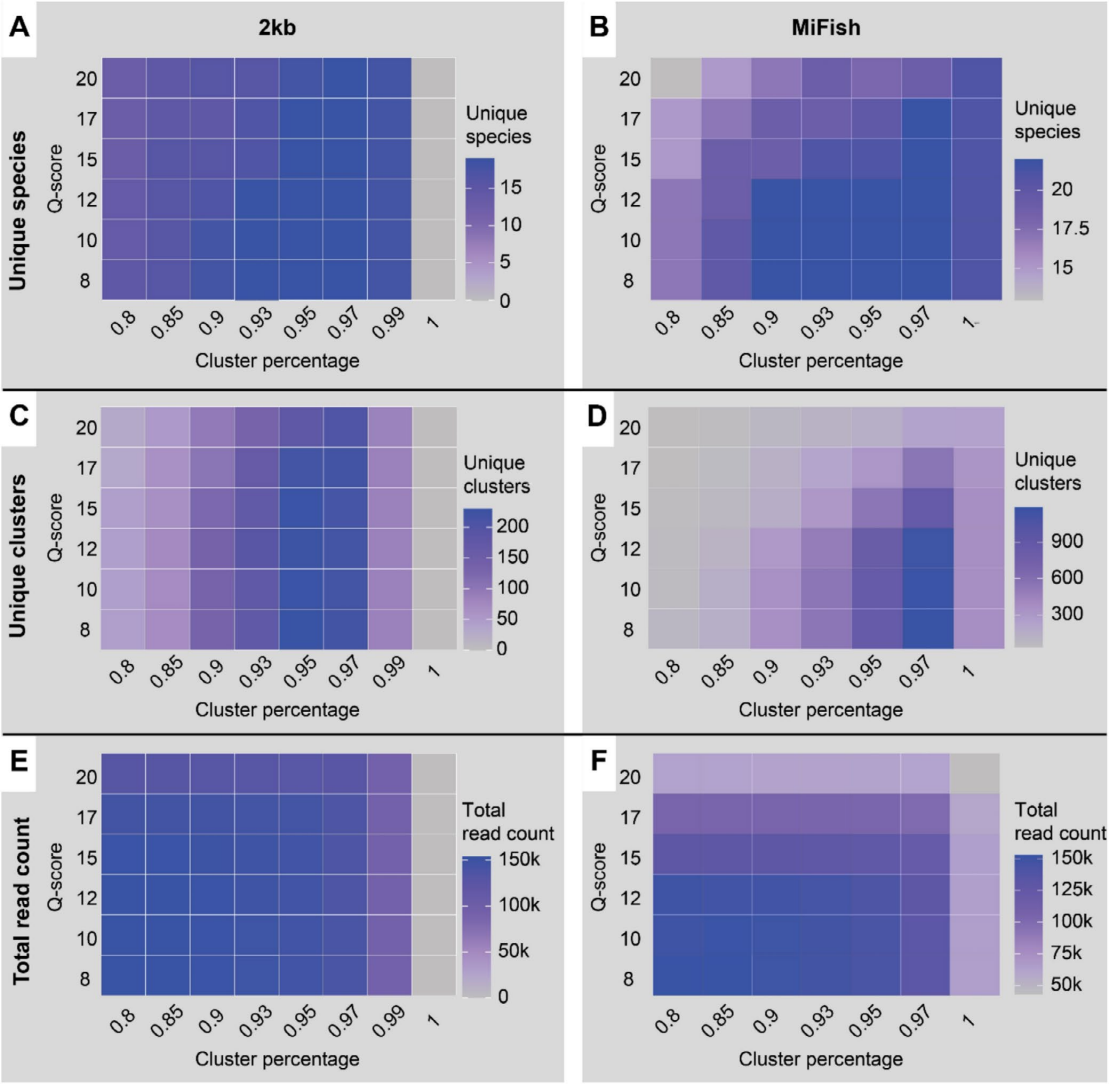
Comparison of DECONA settings for the R10 SUP basecalled reads. The number of unique species that are found when using reads from a certain minimal q‐score (8,10,12,15,17,20) in combination with different minimal % identity threshold values for clusters (cluster percentage, 0.8,0.85,0.9,0.93,0.95,0.97,0.99 (2 kb only) and 1) of (A) 2 kb reads and (B) Mifish reads. Colour gradients indicate the number of species found. Where (C) is the number of unique clusters found with each minimal q‐score in combination with each cluster percentage for 2 kb and (D) MiFish. Colour gradients indicate the number of unique clusters found. (E) The reads that remain with each minimal q‐score in combination with each cluster percentage for 2 kb and (F) MiFish. Colour gradients indicate the total reads found.

Testing of different Q‐score and cluster similarity settings in DECONA shows the optimal settings differ per experimental setup. For the 2 kb amplicon, the number of unique clusters and species is highest with a cluster similarity of 0.95 (clusters and species) and 0.97 (species). Q‐score is of lesser influence, especially when considering unique species (Figure 3A,C). The MiFish amplicon shows the highest number of clusters and species when setting a clustering similarity of 0.97. Also here, Q‐score influences this number to a lesser extent: anything between Q8 and Q17 results in a similar number of clusters or species (Figure 3B,D). The total read count drops for both primer pairs when settings are too stringent (i.e., Q20 or clustering similarity of 1) (Figure 3E,F).

### 3.3 Most Diversity Obtained With Both Primer Pairs in Aquarium Samples, but Species Composition Varies

Sequencing of aquarium samples yielded 529.064 (2 kb) and 220.963 (MiFish) reads, of which 453.714 (2 kb) and 152.395 (MiFish) reads were used for consensus building (Table S9). A barcode distribution of 226.856 ± 160.905 (2 kb) and 76.198 ± 8.010 (MiFish) reads per barcode was achieved. Shapiro–Wilk showed normally distributed data (Shannon: *p* = 0.325, Observed *p* = 0.406) and no significant difference in Shannon index (*t*‐test: *p* = 0.5476) or richness (*t*‐test: *p* = 0.350) although on average more species were found with the 2 kb primer pair (Table S10).

**FIGURE 4:**
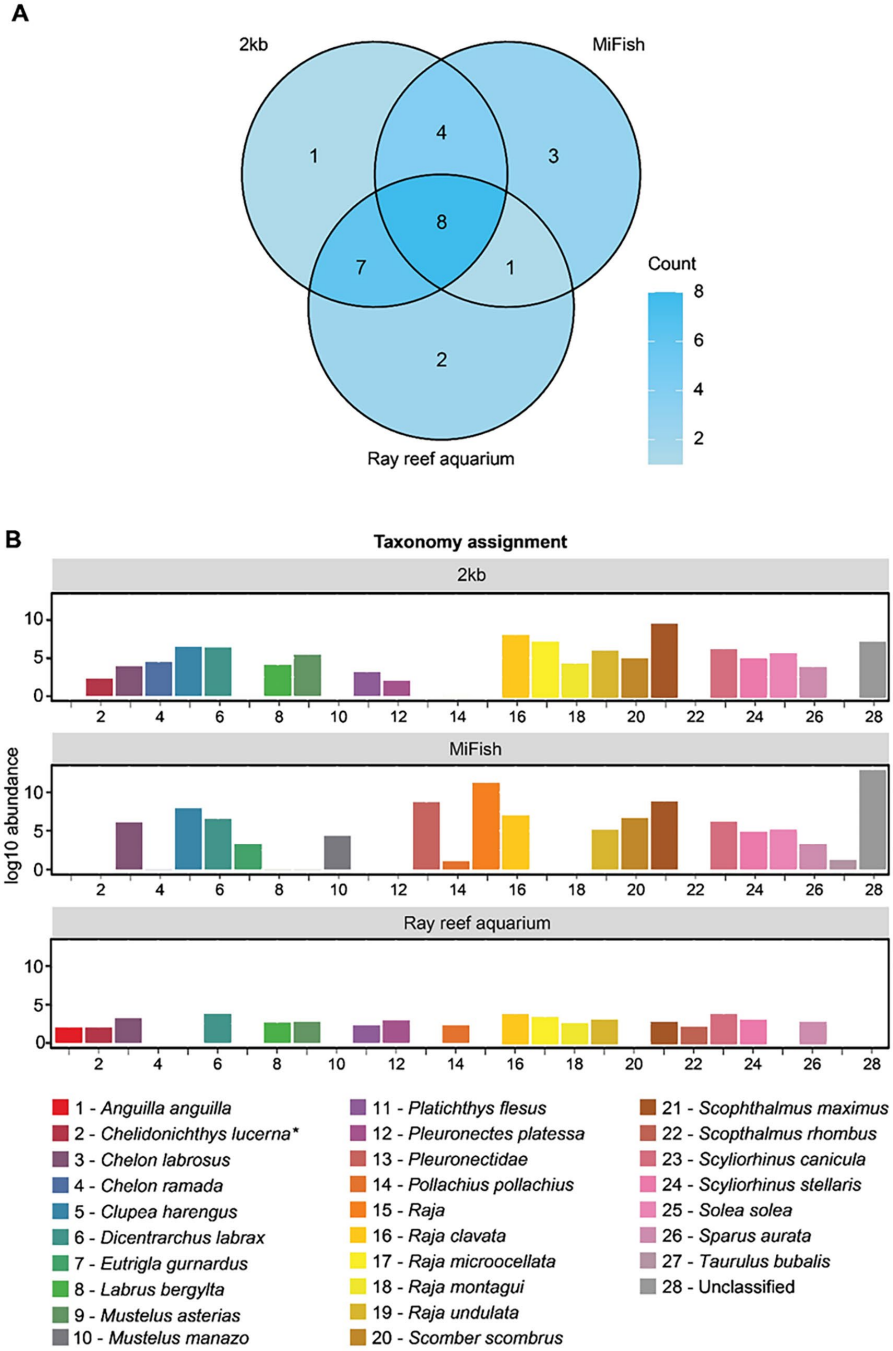
MiFish and 2 kb primer pair in comparison to the species composition of the North Sea Ray Reef aquarium. Taxonomy assignment on species level unless specified otherwise. (A) Venn diagram showing the species overlap in morphological species counts and the primer used (B) Bar plot of the species‐specific differences observed between the actual species present in the Ray aquarium and the results from the analysis using the two different primer pairs.

Analysis of the mock community showed that all species could be detected using the 2 kb Primer pair (Table S14). From the aquarium samples, Eight out of 18 species present in the aquarium could be detected with both primer pairs and an additional seven species could be obtained with 2 kb sequence assignments (Figure 4A). One additional species could be detected with MiFish sequence assignments. Four species were detected with both primer pairs but were not reported as aquarium inhabitants. Sequences from both primer pairs were sometimes incorrectly assigned to a species that belonged to the same genus as the species present in the aquarium (e.g., *Mustelus manazo* , Figure 4B). A total of two species that resided in the aquarium could not be detected in the eDNA samples by either of the primer pairs. These had only one or two individuals in the aquarium of a total of 301 individual fish individuals. *Scopthalmus rhombus* was not present in the reference database of either primer pair. Read count per species and sample can be found in Table S11.

### 3.4 No Significant Difference in Alpha, Beta Diversity in North Sea Field Samples From Different Habitats

**FIGURE 5:**
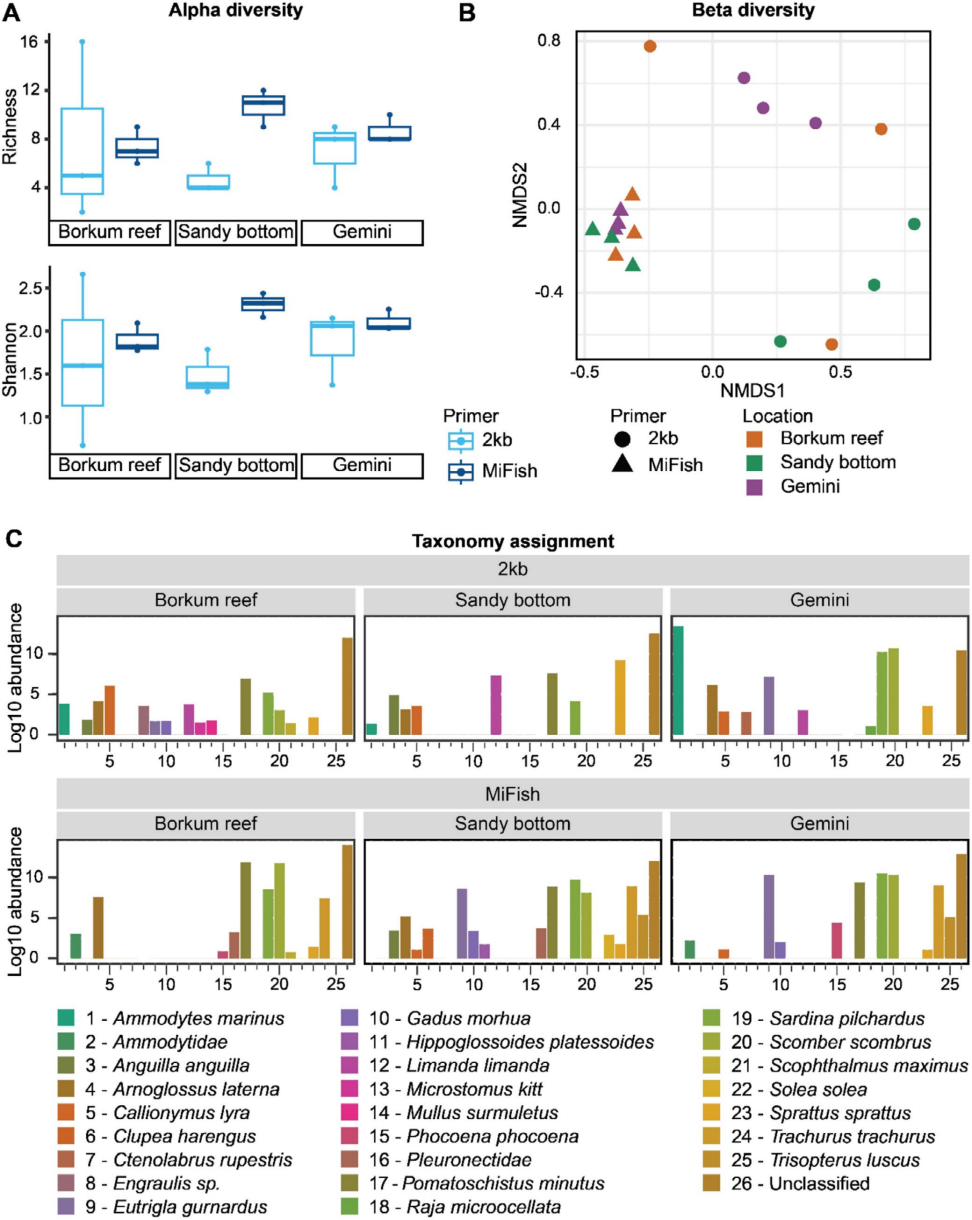
MiFish and 2 kb primer pairs comparison of different eDNA samples taken in the Borkum reef, Gemini wind park and Sandy bottom. Taxonomy assignment on species level. (A) Alpha diversity comparison of richness and evenness, (B) NMDS ordination plot (Bray) showing the similarity between samples. (C) species comparison barplot of the species‐specific differences between the different location and primers.

A total of 1.627.781 (2 kb) and 2.059.490 (MiFish) reads were obtained from the North Sea samples collected at Borkum, Gemini and sandy bottom. 959.538 (2 kb) and 981.304 (MiFish) reads were used for consensus building (Table S9), with a barcode distribution of 106.615 ± (2 kb) and 108.864 ± 88.918 (MiFish) reads per barcode. Samples were normally distributed (Shapiro–Wilk, Shannon index: *p* = 0.401, Observed: *p* = 0.662) and no significant difference in richness was found between primer pairs (Observed, 2‐way ANOVA, *p* = 0151) nor locations (Observed, 2‐way ANOVA: *p* = 0.981) (Figure 5A). Shannon index was also not significantly different between primer pairs (Shannon, 2‐way ANOVA: *p* = 0.069), and between locations (Shannon, 2‐way ANOVA: *p* = 0.7305). The NMDS ordination plot of the beta diversity (Bray Curtis index) shows clustering of sample replicates within location except for the Borkum reef ground processed with the 2 kb primer pair (indicated in colours). Additionally, clustering of primer pairs can be observed within locations (indicated in shapes) (Figure 5B). The effects of location and choice of primer pair were verified with statistical analysis. Homogeneity of variances between samples was found (betadisper: *p* = 0.424) and PERMANOVA showed a significant effect of choice of primer pair (adonis: *p* = 0.001) and location (adonis, *p* = 0.019) and a significant interaction effect (adonis: *p* = 0.031). More details of the statistical results are given in Table S10. Both primer choices showed that unique species were observed with either method. *Clupea harengus* , and *Trisopterus luscus* were only observed using the MiFish primer pair, whereas *Ammodytes marinus* , *Limanda limanda* , *Raja microocellata* and *Ctenolabrus rupestris*are unique for the 2 kb primer pair (Figure 5C and Table S12).

### 3.5 eDNA Samples Taken at Different Shipwrecks Significantly Differ in Alpha and Beta Diversity

**FIGURE 6:**
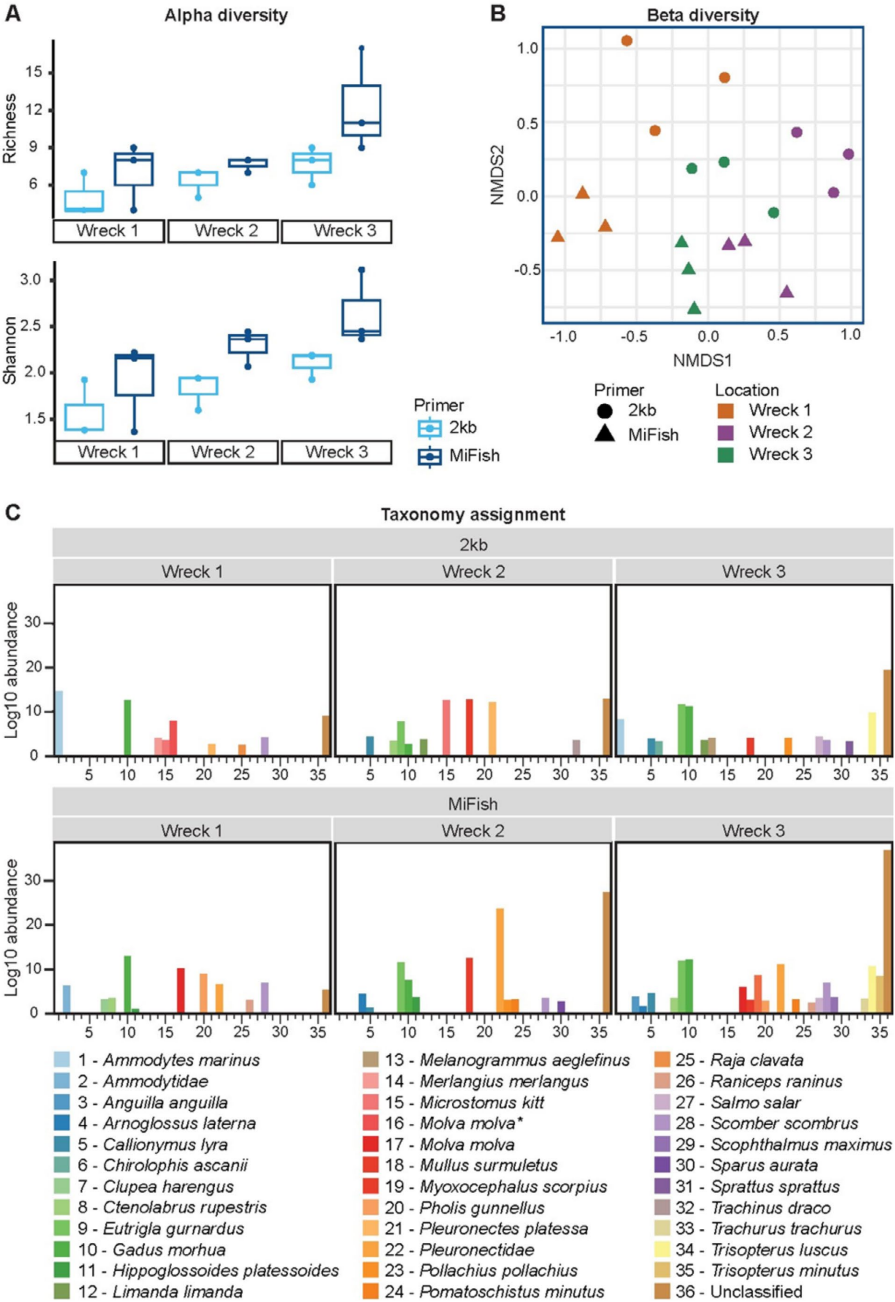
MiFish and 2 kb primer pairs comparison of different eDNA samples taken from different shipwrecks in the North Sea. Taxonomy assignment on species level. (A) Alpha diversity comparison of richness and evenness. (B) NMDS ordination plot (Bray) showing the similarity between samples. (C) Species comparison bar plot of the species‐specific differences between the different location and primers.

Sequencing shipwreck samples resulted in 1.677.936 for 2 kb and 1.301.036 reads for MiFish, of which 1.217.497 (2 kb) and 1.126.749 (MiFish) reads were used for clustering and consensus building (Table S9). A barcode distribution of 186.437 ± 129.453 (2 kb) and 144.559 ± 104.799 (MiFish) reads per barcode was achieved. Shapiro–Wilk showed normally distributed data (Shannon: *p* = 0.605, Observed: *p* = 0.235) and there was a significant difference in richness between primer pairs (Observed, 2‐way ANOVA: *p* = 0.029) and between locations (Observed, 2‐way ANOVA: *p* = 0.026) and no significant interaction effect was found (*p* = 0.435). There was only a significant difference between Wreck 1 and Wreck 3 (Tuckey HSD, *p* = 0.026). A significant difference in Shannon index was observed between primer pairs (Shannon index, 2‐way ANOVA: *p* = 0.010) and between locations (Observed, 2‐way ANOVA: *p* = 0.014) and no significant interaction effect was found (*p* = 0.8705, Figure 6A). There was only a significant difference in Shannon index between wreck 1 and 3 (*p* = 0.011). The NMDS ordination plot shows clustering between wrecks (indicated in colours) but also between primer pair (indicated in shapes) (Figure 6B). PERMANOVA showed a significant difference in beta diversity between wrecks (PERMANOVA, *p* = 0.001) and Primer (PERMANOVA, *p* = 0.001) and an interaction effect was also observed (*p* = 0.007). Samples were homogeneous (betadisper, *p* = 0.575). Nevertheless, overall, the species compositions were consistent between primer choice in each location, albeit MiFish detected more species. In addition, both primer pairs picked up unique species where the 2 kb primer pair for example identified, *Melanogrammus aeglefinus* , *Merlangius merlangus* and *Limanda limanda* . MiFish on the other hand had unique findings of *Clupea harengus* , *Anguilla anguilla* , and *Trachurus trachurus* (Figure 6C and Table S13).

## 4 Discussion

With the rise of Oxford nanopore sequencing, it now becomes increasingly feasible to use long read sequencing in metabarcoding studies. We introduced and tested the settings of the bioinformatics pipeline DECONA that enables processing of raw read Nanopore data to species assignment with just one line of code. In addition, we explored the utility of a longer amplicon fragment by comparing it to a commonly used short amplicon. Utility was tested by using an *in silico* approach, and subsequently, we tested the primer pair on samples from an aquarium with a known species composition as well as on field samples. We showed that the DECONA pipeline improves the accuracy of Nanopore reads to enable species level assignment and that the most optimal settings for DECONA depend on methodological choices. We also showed that the designed primer pair is not only bony fish specific, but also targets elasmobranchs. In sillico results show that longer target fragments can help increase correct species level assignments for genetically closely related species. This is also reflected in the results from the Aquarium samples: *Platichthys flesus* and *Pleuronectus platessa* are both detected with the 2 kb primer pair but cannot be identified to species level with MiFish primer pair and are listed as *Pleuronectidae*. The same is true for several *Raja* species. Aquarium samples also showed that most of the species were shared between both amplicon fragment lengths but that more species could be correctly identified with the 2 kb primer pair, mainly due to improved species level assignment. In the field samples, the alpha diversity was generally higher for the short fragment size, possibly due to lower abundance of longer fragments in the environment. Nevertheless, we have demonstrated that it is possible to use long and short amplicons for species level eDNA metabarcoding using Nanopore sequencing.

Although ONT based long read metabarcoding is shown to be successful in bacterial studies (Krehenwinkel et al. 2019; Matsuo et al. 2021), there is a limited number of reports that validated its use for marine biodiversity studies (Semmouri et al. 2021). Thus, these results help build a foundation to further study the added value of long read sequencing in marine vertebrate biodiversity assessments. Nevertheless, it remains challenging to adequately demonstrate possible strengths of long read amplicons sequencing as methodological choices are often different between studies (Ruppert et al. 2019; Wang et al. 2021). Also in this study, different methodological choices were made, including a limited number and different approaches of sampling, as offshore marine conditions often restricted effective sampling efforts. Nevertheless, studies that address longer fragments, especially with diluted, environmental samples will improve the understanding of how long amplicons may enhance eDNA based marine biodiversity assessments.

### 4.1 DECONA Enhances Read Accuracy and Species Assignment, but Settings Should be Tailored to the Experiment at Hand

As it was possible to adequately assign reads to species level, the developed sequence read processing pipeline DECONA enables a consensus sequence accuracy as species level assignments were accepted from 98%, which is comparable to Illumina read accuracy (Caporaso et al. 2011). There is limited automated bioinformatics processing reported in Nanopore based studies, especially for metabarcoding (Santos et al. 2020). This study introduces the DECONA pipeline (https://github.com/saskia‐oosterbroek/DECONA), thereby contributing to the availability of bioinformatics software to process Oxford Nanopore sequence output. Once installed, one line of code suffices to correctly run the pipeline and enables data processing for scientists with limited experience with the command line. The bioinformatics tools integrated in DECONA are well established programs in genomics and transcriptomic studies. For example, tools such as CD‐Hit (Huang et al. 2010) have previously been used in the analysis of Nanopore sequence data for clustering and consensus building of fish amplicon‐based sequences (Voorhuijzen‐Harink et al. 2019). Reference based polishing was successfully applied when identifying benthic organisms on autonomous reef monitoring structures (Jin et al. 2020) using *minibarcoder.py* (Srivathsan et al. 2018). The combination of both clustering and *de novo* alignment‐based polishing with racon (Vaser et al. 2017) and medaka (https://nanoporetech.github.io/medaka/) has previously been used for the correction of metagenomes (Rodríguez‐Pérez et al. 2020). In contrast, the DECONA pipeline combines similarity‐based clustering based on short word tables instead of an alignment approach in combination with alignment‐based polishing with racon and medaka, which further increases the sequence accuracies. Limitations of DECONA may lie in the necessity to cluster, which makes it possible that reads from genetically similar organisms end up in the same clusters, resulting in lower detection sensitivity than is actually sequenced. In addition, clustering with DECONA also disregards singletons, as such missing the rare reads in datasets. Fortunately, due to the fast development of Oxford Nanopore sequencing technologies, new sequencing chemistries with reduced sequencing error rates and basecalling algorithms are often released and accuracy is now at a 99.8% raw read accuracy for model organisms (Srivathsan et al. 2021). Therefore, by using new chemistries it may become possible to skip the clustering and polishing process altogether and perform raw read identification using amplicon sequence variants (ASVs) in the near future, as is the standard for Illumina platforms (Van Der Reis et al. 2022).

Choices in bioinformatics influence the results and affect conclusions that can be drawn. Settings in DECONA should therefore be carefully considered and especially cluster similarity is of importance. Setting a high cluster similarity result in more clusters, but with the risk of obtaining more singletons that will then be discarded. Setting a low cluster similarity causes similar reads to be clustered together, reducing the observed diversity of a sample and especially prone to happen when closely related species are expected in the sample. The optimal cluster similarity changes with amplicon length. Therefore, it is important to test several settings of DECONA on a subset of each dataset to validate which settings give the most sensible results for the amplicon (length) of choice as well as the sequencing chemistry that is used.

### 4.2 In silico Identification Shows Increased Species Level Identification Using Longer DNA Fragments

Alignment of species within the *Pleuronectidae, Ammodytidae* and *Raja* families showed a genetic variability insufficient to differentiate related species when aligning the MiFish target fragments. These assignment problems have already been reported for North Sea fish species (e.g., Barco et al. 2022). The 2 kb target fragment alignment shows that for some species indeed the sequence dissimilarity increases to up to 2%. However, for *Hyperoplus lanceolatus* and *Ammodytes tobianus* , it remains impossible to distinguish on species level on the complete 2 kb target region, which demonstrates that for some species it is needed to use an additional target region to adequately assign on species level. In addition, for *Pleuronectus platessa* and *Platichthys flesus* there are an additional 11 nucleotide differences found on the 2 kb target region. The *in silico* comparison therefore shows that taxonomic identification to species level can be obtained when using long read metabarcoding, but that there is a need to consider other fragments and lengths to be able to differentiate between genetically highly similar species.

### 4.3 Assignments From the Aquarium Samples Show Overlap Between Primer Pairs, Primer Pair Specific Differences, and False Positives

Most species in the aquarium could be detected with both primer pairs used. The species *Scomber scombrus* was detected by both primer pairs but did not live in the aquarium. This species was used as feed for the different piscivorous animals in the aquaria, showing that both primer pairs are also able to pick up the signal of animals that are only temporarily present as part of the diet of the aquarium inhabitants (personal communication P. Bunskoek, Dolfinarium Harderwijk).

Analysis of the eDNA aquarium samples also detected unique species for each primer pair. For example, the sequences from the 2 kb primer pair better represent the different ray and *Pleuronectidae* species, whereas the MiFish primer pair could capture DNA from *Pollachius pollachius* . As the 2 kb primer pair was designed in such a way that it should amplify all *Gadiformes* species (See S3, Supporting Information S1), this finding suggests that, using multiple markers would improve the detection of the present species, and agrees with earlier findings illustrating that multiple markers give a better representation of the complete biodiversity (Cordier et al. 2019). For both primer pairs there is also false positive species assignment as is illustrated with *Mustelus mananzo*. Despite the careful choices made in this study for correct species identification, false positive species assignments can still arise. This may occur, since BLASTn assigns reads to top hit species while there may also have been a similar match with the correct species, but with a smaller alignment length. Taxonomy assigners that are currently used for Illumina MiFish metabarcoding make use of Naïve Bayesian classifiers such as RDP (Cole et al. 2003) that can do quick taxonomic assignment for ASV metabarcoding sequences and also assigns taxa to a higher taxonomic level when there are sequences with multiple hits. For Nanopore based consensus sequences such assigners are, to our knowledge, not yet applied. Therefore, it is still needed to manually adjust results based on a priori knowledge on the genetic similarity, despite thorough ruling‐out of such events in data‐processing.

There were also species present in the aquarium that were not detected by any primer pair. It is often observed that eDNA methods do not identify the complete biodiversity, despite using a multi‐marker approach (Morey et al. 2020). *Scophthalmus rhombus* for example was not detected by any molecular method as there is no representation of its 12‐16S fragment in public databases, making it impossible to assign a read to this species. This further stresses the need to continue improving genetic reference databases both with short fragments as well as for complete (mitochondrial) genomes. Of the undetected species only one or two individuals were present in the aquarium, which suggests that the lack of detection is a result of low initial DNA concentration of those species. And since there are a total of 301 specimens in total in the aquarium (S2), it is possible that that the overrepresentation of DNA of other species have masked these detections. This is in line with inconsistent detection of rare taxa between filters described in previous reports (Evans et al. 2016; Kelly et al. 2014; Morey et al. 2020), and species detection could be improved by using more replicates (Beentjes et al. 2019; Evans et al. 2016) or collecting a larger volume of water where possible. An alternative explanation for the lack of detection of these low abundant species could come from the sequence processing. As it is necessary to cluster raw reads, rare reads can end up as singletons or in a cluster that is removed during further sequence processing. Overall, despite the detection of false positives, false negatives, and primer pair specific results, both primer pairs, and especially the 2 kb primer pair could identify the majority of the marine vertebrates, identifying an additional 7 species, mostly due to increased possibility for species level identification.

### 4.4 Field Samples Show Lower Diversity in 2 kb Fragment Length

The alpha diversity in both Shannon index and richness was overall higher for the MiFish results in the in the field samples, although not always significant. An explanation for the increase in alpha diversity could lie in the eDNA fragment length sampled. In aquaria it can be expected that the relative concentration of eDNA in the water and especially of freshly released long eDNA fragments in the water is high, hence more diversity could be found with the 2 kb primer pair in the aquaria. Finding a lower alpha diversity in the field samples may be due to lower fish density, and potentially faster breakdown of free extracellular DNA (Seymour et al. 2018). Therefore, it is likely that the average size of DNA fragments present in the field eDNA samples is smaller and hence a smaller proportion can be successfully amplified with the 2 kb primer pair, while amplification of short DNA fragments with the MiFish primer pairs is still possible. This is in line with the hypothesis that longer fragments of nucleic acids in the environment break down rapidly, and that therefore longer fragments be used to incorporate time‐scale information into the eDNA analysis (Jo 2023). Our results thus suggest that the MiFish primer pair approach can identify additional signals from taxa that released their DNA longer ago, while the 2 kb primer pair would provide temporal snapshots of species that have been present more recently. There is a need to further assess how DNA length is affected by degradation both intracellularly and extracellularly (Jo 2023) to understand how read lengths could be exploited to obtain additional insight into diversity on a temporal scale.

Additionally, the species compositions between the locations and primer pair used was in most cases consistent between replicates (S13) which was especially apparent in local wreck sampling. In addition, the wreck samples were more consistent in terms of species compositions between primer pairs, as most of the species could be found with either primer pair, despite using a sub optimal filed control. Primer specific observations, as consistent detections of *Limanda limanda* and several *Gadiformes* species as *Merlangius merlangus* and *Melanogrammus aeglefinus* , seem specific to the 2 kb primer pair, likely because these species are genetically too similar for correct species level assignments with MiFish. This consistency was less obvious, however, in the Gemini/Borkum dataset and, especially with the 2 kb primer pair. Different sampling methods have resulted in these findings. The wreck samples were taken while diving, which may have provided a more stable water column, allow for collection on a precise location on these local biodiversity hotspot (Fowler and Booth 2012) whereas the Gemini/Borkum samples were taken less locally using a niskin bottle. Alternatively, due to the several rounds of revisions that this work has undergone, DNA extracts were partly re‐analysed years later (see Supplementary text 1) and may have resulted in degradation of the sample over time. Nevertheless, Nanopore based long read metabarcoding in combination with read processing with DECONA, can be utilised to find differences in diversities between ecologically relevant sites, albeit with lower alpha diversities than with MiFish primer pair. Thus, by carefully choosing the settings in DECONA, the combination of long and short reads enables assessing the fish biodiversity on species level at multiple different sample sites (e.g., shipwreck sampling), where short reads enhance detected alpha diversity and long reads additionally provides a species level assignment of genetically closely related species while possibly providing a temporal snapshot of the community.

## 5 Conclusion

This study demonstrates and validates an eDNA metabarcoding approach using Nanopore long read technology. To enable this approach, we present our Nanopore sequence processing pipeline DECONA. DECONA is bioinformatics pipeline that allows researchers to set the right cluster similarity and can be tailored to the amplicon length and ONT chemistry at hand. We demonstrate an increased species resolution due to the longer DNA fragments analysed. We further show limitations such as false positive assignments and limited detection of rare species suggesting the importance of using multiple markers for increased detection resolution for fish. Further research should focus on exploring the use of long read metabarcoding to gain biodiversity information on a spatial–temporal scale to further understand the role of long reads for eDNA biodiversity assessments. In addition, studies should focus on the possibility to use Nanopore generated raw reads directly, to further implement Nanopore based (long read) metabarcoding as standard to the molecular ecology toolbox. Moreover, it is essential that addition of longer reference sequences to databases, preferably of full (mitochondrial) genomes, maintains a high priority in marine molecular ecology. Only then can long read based DNA metabarcoding and metagenomics develop to its full potential to serve as monitoring tool.

## Author Contributions

Karlijn Doorenspleet, Lara Jansen and Reindert Nijland designed the experiment. Lara Jansen, Pauline Kamermans, Oscar Bos, Reindert Nijland, Albertinka Murk and Erik Wurz were involved in sample collection and processing. Karlijn Doorenspleet, Lara Jansen and Reindert Nijland did the laboratory work. Saskia Oosterbroek, Karlijn Doorenspleet and Reindert Nijland designed the bioinformatics pipeline DECONA. Karlijn Doorenspleet and Reindert Nijland conducted the data analysis. Karlijn Doorenspleet, Lara Jansen, Saskia Oosterbroek, Reindert Nijland and Albertinka Murk interpreted the data; all authors wrote and revised the manuscript.

## Conflicts of Interest

R.N. has received reimbursement for travel, accommodation and conference fees to speak at events organized by Oxford Nanopore Technologies. The remaining authors declare no conflicts of interest.

## Supporting information


Appendix S1:
**Table S1:** The total amount of reads and species found for each control sample.
**Table S2:** The species that are present in the ray reef aquarium.
**Table S3:** The species of the North Sea and their representation of reference fragments present in the used database.
**Table S4:** accession numbers of NCBI references for in silico comparison.
**Table S5:** BLAST database entries North Sea.
**Table S6:** Clusters that are assigned to multiple species with the same e‐value (> 98%) per primer pair and cluster similarity setting, Aquarium samples.
**Table S7:** Rarefaction curves.
**Table S8:** Off target hits from the different datasets.
**Table S9:** Read counts after processing for each dataset.
**Table S10:** Statistical analysis.
**Table S11:** Read count per species and per sample from the aquarium dataset.
**Table S12:** Read count per species and per sample from the Borkum/Gemini dataset.
**Table S13:** Read count per species and per sample from the shipwrecks dataset.
**Table S14:** Mock community findings and read counts.

Appendix S2:

## Data Availability

All sequence data used for the writing of this manuscript have been uploaded to the European Nucleotide Archive (ENA) under project accession PRJEB81670. The code for the DECONA pipeline is available through https://github.com/Saskia‐Oosterbroek/decona and the scripts used to process the data can be viewed and downloaded via https://github.com/karlijn‐doorenspleet/decona‐postprocessing/.Benefits from this research increase from sharing our pipelines, data and results on public databases as described above.
