# Supplementary material for: The Long and the Short of It: Nanopore‐Based eDNA Metabarcoding of Marine Vertebrates Works; Sensitivity and Species‐Level Assignment Depend on Amplicon Lengths
Source: Mol Ecol Resour. 2025 Feb 10;25(4):e14079. doi: 10.1111/1755-0998.14079 (PMC11969631; doi:10.1111/1755-0998.14079)
Supplement: Supplementary file 2 — Appendix S2: [file MEN-25-e14079-s001.docx]

Supplementary text 1: Redesign of the forward primer

One of the reviewers discovered a mismatch in the last nucleotide of the 3` side (T instead of C) of the forward primer targeting the 12S region for several Species in the order of Gadiforme. This may have resulted in the underrepresentation of several Species such as *Trisopterus* species, *Gadus morhua*, *Merlangus merlangus*, several *Molva* species, *Melanogrammus eaglefinus*, *Brosme brosme*, *Boreogadus saida*, *Ciliata mustela*, *Enchelyopus cimbrius*, *Gaidropsus vulgaris*, *Gadiculus argenteus*, *Merluccius merluccius*, *Lampris guttatis*, *Zeus faber*, *Mustelus mustelus*. According to the reviewer, this is likely a critical mismatch, causing the underestimation or a total lack of amplification of these species, hindering to report the correct community analysis. Previous versions of this manuscript, available via biorxivs, do not describe the right primer and as a result, a study used the previous primer and showed it failed to detect several Gadiformes species in a mock community (Maggini et al., 2024, supplementary material). For this revised manuscript, we redesigned the forward primer and re-analyzed the samples with this primer. To further clarify the improved performance of this primer, we compared the performance with the previous version in this supplementary material.

As stated in the main text for the design of the new forward primer, all available mitochondrial and 12S-16S fragments were used that were available in NCBI (visited September 2024). To improve detection of the Gadiformes, the existing C on the 3’ end was replaced with a degeneracy (N). In addition, another degeneracy (Y) replaced the nucleotide on the second position of the 3’ end of the reverse primer, to also improve the detection of the Petromyzontiformes (**Table 1**).

Comparison with the newly designed 2kb primer showed that indeed there is an increased amount of Gadiformes species found in all datasets. For the aquarium samples, additional species were found as *Mustelus asterias* and *Labrus bergylta* that were previously not found (**Figure 3b**). Samples from the North Sea dataset also showed the detection of *Gadus morhua* where this was not detected with the old primer version (Figure SXa). *Trisopterus luscus* on the other hand could not be detected, despite findings of this species using the MiFish primers (**Figure 4c**). Nevertheless, using the new primer pair for the wreck samples of the North Sea (**Figure 5c**), *T. luscus* was detected, as many other Gadiformes such as *Melanogrammus aeglefinus*, *Merlangius merlangius*, *Gadus morhua*, *Pollachius Pollachius*, and the detection of *Molva sp*. indicating that the primer improves the detection of several Gadiformes species (**Figure S2b**). However, both primers identified additional Species that were unique for each primer pair design (**Figure S1, S2b**) and each design also showed primer specific clustering, although the distances between clusters that represented different locations remained (**Figure S2a**).

This comparison shows that, the new primer pair improves the detections of Gadiformes, and other species are likely better represents the community as *G. morhua*, *Trisopterus luscus*, Molva sp. as well as *Mustelus asterias* and others were detected in aquarium and field samples. Therefore, problem in detection as pointed out by the reviewer and reported by Maggini et al. (2024) is likely to be resolved. Thus, these data show that the newly designed primer pair is preferred over the pair from the previous version. However, also primer specific community was found, from unique findings in both primer pairs. Although only minimal changes were made to the original primers, it is possible that the number of degeneracies resulted in different annealing affinities of on target and off-target sequences (Yang et al., 2023). In addition, other processing biases such as PCR stochasticity, handling biases, and differences in the ever updating chemistry and bioinformatics may also play a role (van der Loos & Nijland, 2021). Lastly , the dataset for this recent revision was several years after the previous attempts and, as longer fragments are more easily degraded and less likely to be present (Jo, 2023) it is possible that degradation of the longer fragment in the DNA extract itself may have resulted in these primer specific findings. Nevertheless, overall species level detection was improved, further emphasizing the potentials of long read metabarcoding for vertebrate biodiversity assessment studies.

Reference:

Jo, T. S. (2023). Utilizing the state of environmental DNA (eDNA) to incorporate time-scale information into eDNA analysis. *Proceedings of the Royal Society B: Biological Sciences*, *290*(1999), 20230979. https://doi.org/10.1098/rspb.2023.0979

Maggini, S., Jacobsen, M. W., Urban, P., Hansen, B. K., Kielgast, J., Bekkevold, D., Jardim, E., Martinsohn, J. T., Carvalho, G. R., Nielsen, E. E., & Papadopulos, A. S. T. (2024). Nanopore environmental DNA sequencing of catch water for estimating species composition in demersal bottom trawl fisheries. *Environmental DNA*, *6*(3), e555. https://doi.org/10.1002/edn3.555

van der Loos, L., & Nijland, R. (2021). Biases in bulk: DNA metabarcoding of marine communities and the methodology involved. *MOLECULAR ECOLOGY*, *30*(13), 3270–3288. https://doi.org/10.1111/mec.15592

Yang, J., Zhang, L., Mu, Y., & Zhang, X. (2023). Small changes make big progress: A more efficient eDNA monitoring method for freshwater fish. *Environmental DNA*, *5*(2), 363–374. https://doi.org/10.1002/edn3.387


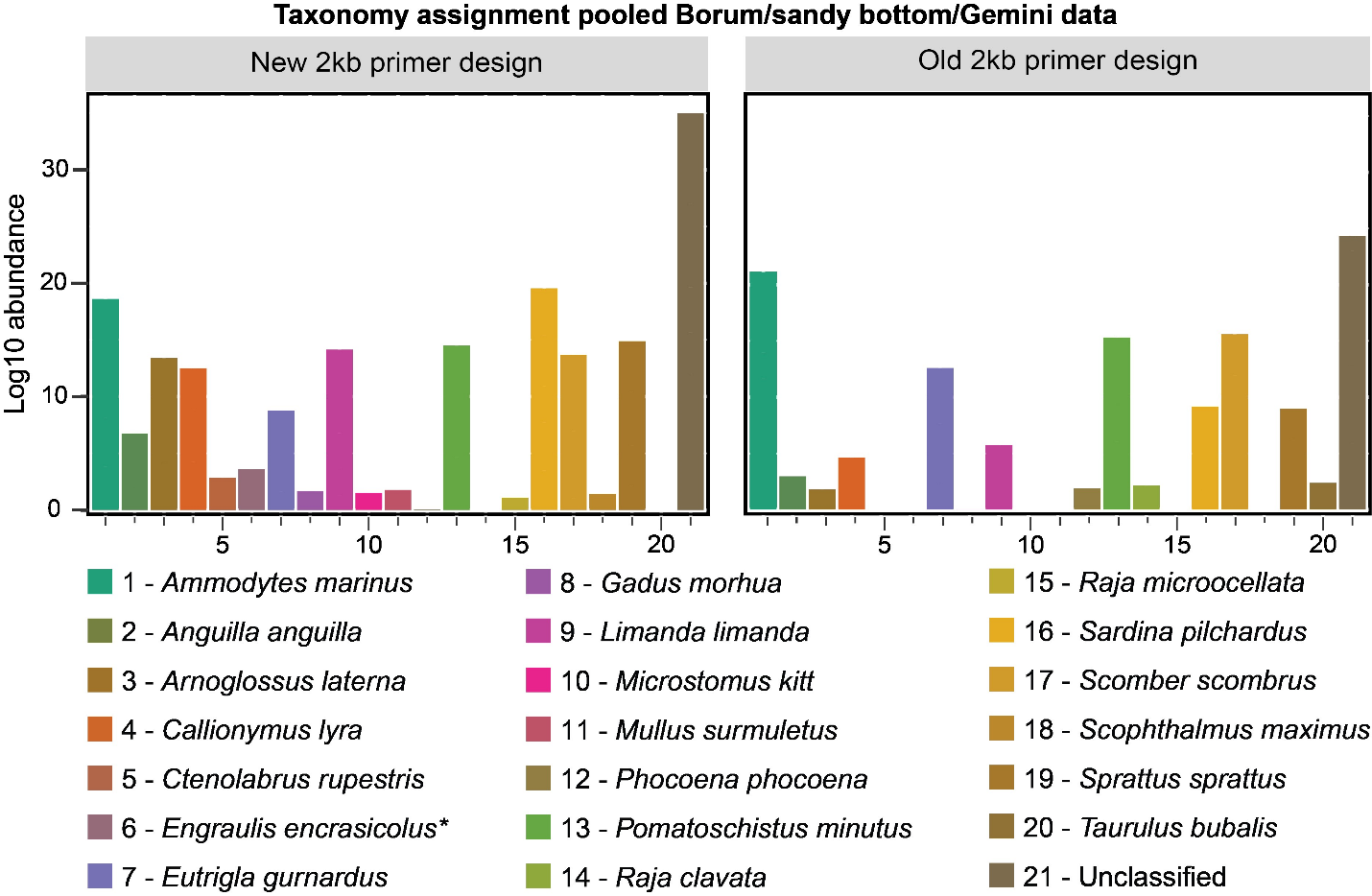


**Figure S1**: comparison of detected species (colored bars) found (summed log10 read abundance) of all samples with the new 2kb design vs. the old 2kb design in the Borkum .gemini dataset. The figure shows that the newly designed primer pair overall picks up more species, and both primer pairs detected unique species.


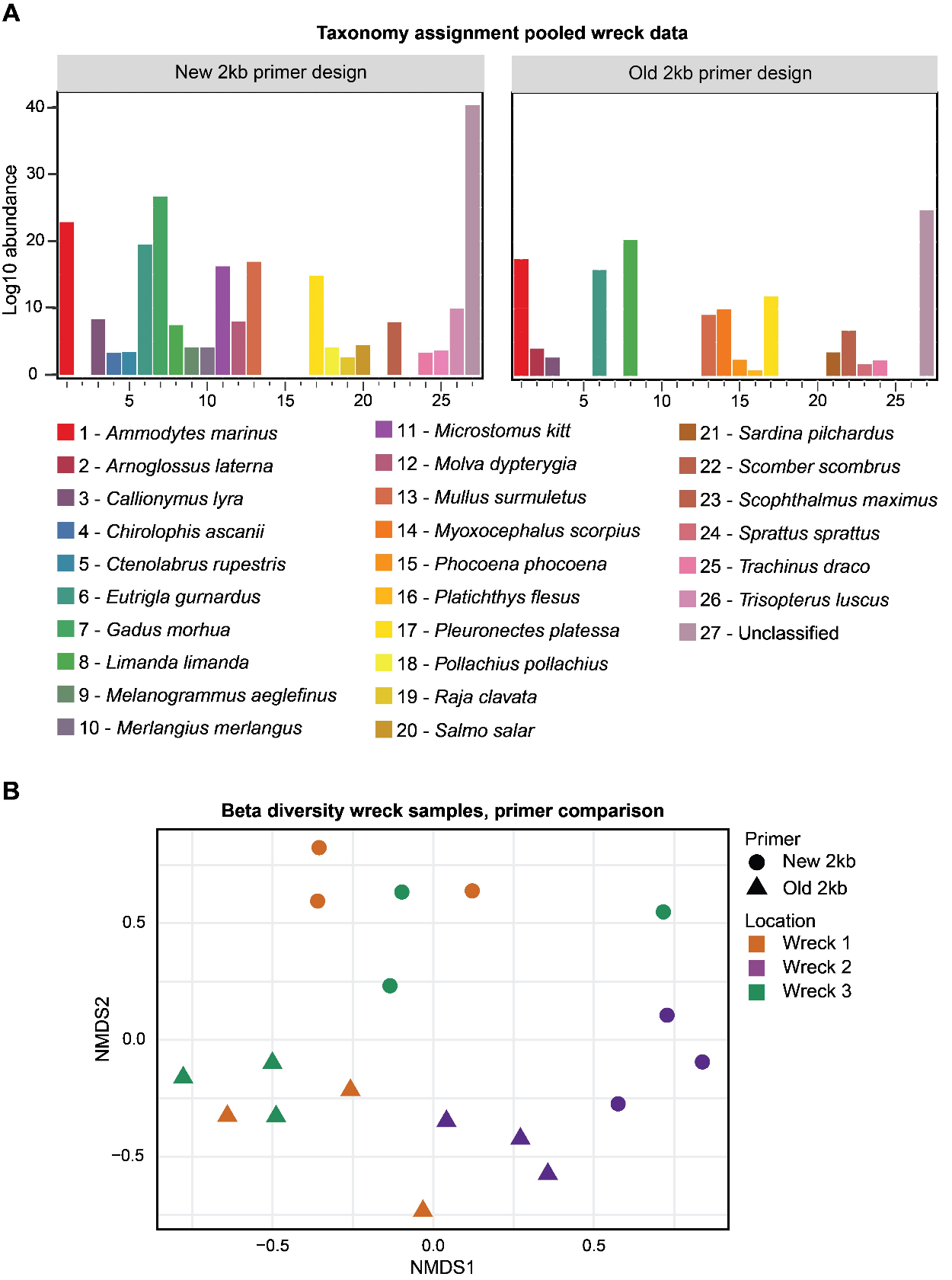


**Figure S2**: A) Comparison of detected species (colored bars) found (summed log10 read abundance) of all samples with the new 2kb design vs. the old 2kb design in the Wreck dataset. The figure shows that the newly designed primer pair overall picks up more species, and both primer pairs detected unique species. B) Ordination using Nonmetric multidimensional scaling (NMDS) for the wreck samples between each location (colors) and each primer design (shapes).
